# Multivariable MR Can Mitigate Bias in Two‐Sample MR Using Covariable‐Adjusted Summary Associations

**DOI:** 10.1002/gepi.22606

**Published:** 2025-01-15

**Authors:** Joe Gilbody, Maria Carolina Borges, George Davey Smith, Eleanor Sanderson

**Affiliations:** ^1^ MRC Integrative Epidemiology Unit University of Bristol Bristol UK; ^2^ Population Health Sciences University of Bristol Bristol UK

## Abstract

Genome‐wide association studies (GWAS) are hypothesis‐free studies that estimate the association between polymorphisms across the genome with a trait of interest. To increase power and to estimate the direct effects of these single‐nucleotide polymorphisms (SNPs) on a trait GWAS are often conditioned on a covariate (such as body mass index or smoking status). This adjustment can introduce bias in the estimated effect of the SNP on the trait. Two‐sample Mendelian randomisation (MR) studies use summary statistics from GWAS estimate the causal effect of a risk factor (or exposure) on an outcome. Covariate adjustment in GWAS can bias the effect estimates obtained from MR studies conducted using covariate adjusted GWAS data. Multivariable MR (MVMR) is an extension of MR that includes multiple traits as exposures. Here we propose the use of MVMR to correct the bias in MR studies from covariate adjustment. We show how MVMR can recover unbiased estimates of the direct effect of the exposure of interest by including the covariate used to adjust the GWAS within the analysis. We apply this method to estimate the effect of systolic blood pressure on type‐2 diabetes and the effect of waist circumference on systolic blood pressure. Our analytical and simulation results show that MVMR provides unbiased effect estimates for the exposure when either the exposure or outcome of interest has been adjusted for a covariate. Our results also highlight the parameters that determine when MR will be biased by GWAS covariate adjustment. The results from the applied analysis mirror these results, with equivalent results seen in the MVMR with and without adjusted GWAS. When GWAS results have been adjusted for a covariate, biasing MR effect estimates, direct effect estimates of an exposure on an outcome can be obtained by including that covariate as an additional exposure in an MVMR estimation. However, the estimated effect of the covariate obtained from the MVMR estimation is biased.

## 1 Introduction

Mendelian randomisation (MR) uses the properties of germline genetic variants to strengthen inference regarding the influence of potentially modifiable exposures on disease (Davey Smith and Ebrahim 2003; Sanderson et al. 2022). MR can be implemented as an instrumental variables (IV) analysis, using the genetic variants as instruments. MR requires a set of assumptions to obtain reliable estimates of the effect of the exposure on the outcome. First, that the genetic variants used as instruments are associated with the risk factor of interest; second, that there are no unmeasured confounders of the association between the genetic variants and outcome; and third, that the genetic variants act on the outcome only through the risk factor of interest (Richmond and Davey Smith 2022a). A fourth assumption is required for the interpretation of the magnitude of effect sizes (Sanderson et al. 2022). One such assumption is that there is a monotonic association between the instrument and the exposure (Sanderson et al. 2022; Labrecque and Swanson 2018). An alternative version of this assumption is of no simultaneous heterogeneity, that is, that any heterogeneity in the association between the instrument and exposure is unrelated to any heterogeneity between the exposure and outcome (Hartwig et al. 2020). Throughout this paper, we assume monotonicity and interpret the results obtained under this assumption.

MR can be performed using summary data from GWAS by combining single‐nucleotide polymorphism (SNP)‐exposure and SNP‐outcome associations identified through genome‐wide association studies (GWAS) to estimate the causal effects of the exposure on the outcome (Sanderson et al. 2022; Richmond and Davey Smith 2022). GWAS use a hypothesis‐free methodology and test the association of hundreds of thousands of SNPs with a risk factor in large populations to identify the particular SNPs associated with that risk factor (Manolio 2010; Burgess, Dudbridge, and Thompson 2016). As each variant explains a relatively small amount of the variation of a trait, large sample sizes are often required to have sufficient power for MR effect estimation. Current large GWAS either use large datasets like UK Biobank, which contains over 500,000 individuals (Sudlow et al. 2015) or the very largest GWAS use groups of biobanks and have sample sizes of well over 1 million (Liu et al. 2019; Okbay et al. 2022). However, many GWAS studies available have been conditioned on a covariate to improve the power of the study to identify variants. For example, cardiometabolic traits such as blood pressure are often adjusted for body mass index, while a trait such as lung function may be adjusted for height and smoking status (Evangelou et al. 2018; Soler Artigas et al. 2015).

These covariate adjustments can introduce bias into the estimated SNP trait associations (Aschard et al. 2015). The adjustment for the covariate creates a path between the genetic variant and the trait due to the unmeasured common genetic or environmental cause between the covariate and the trait and so causes an association to be identified that is not due to a true association between the SNP and trait (Cole et al. 2009). This alters the set of SNPs that are identified by the GWAS as associated with the trait as SNPs not associated with the trait of interest may be identified by the GWAS as associated with that trait. Second, when an unmeasured genetic or environmental risk factor is shared between the covariate and the trait of interest the covariate acts as a collider in the path between the genetic variant (*g*) and the trait of interest leading to biased estimates for the association between the genetic variant and the trait of interest.

Previous simulation studies have shown that the use of covariate‐adjusted GWAS in MR analyses can bias effect estimates in ways that are difficult to predict and that are highly dependent on the underlying (and unobserved) causal structure (Hartwig et al. 2021; Walker et al. 2021). In simulations where no unobserved common causes of the exposure and outcome in the MR study exist the covariate adjustment eliminates bias in the presence of pleiotropic effects of the SNP on the covariate. However, the existence of unobserved confounders is one of the main motivations for performing MR. In simulations where unobserved confounding was present covariate adjustment was shown to lead to bias, particularly when there are also confounders of the covariate and the outcome in the MR study (Hartwig et al. 2021). The lack of alternative data means many MR studies have used adjusted summary results previously. For example, such data has been used while investigating the effect of adiposity or waist‐to‐hip ratio on cardiometabolic traits (Dale et al. 2017; Emdin et al. 2017). As highlighted above the use of such data will lead to biased MR estimates and potentially incorrect inference about the causal effects of the traits included in those analyses.

When the GWAS for the exposure of interest has been adjusted for a covariate the effect estimate obtained is the effect of the genetic variant g on the exposure X conditional on the covariate C. (Aschard et al. 2015) show that for a single genetic variant this can be approximated to;

$${\hat{\pi }}_{A}\approx (-{\hat{\pi }}_{C}\rho_{\mathrm{CX}}+{\hat{\pi }}_{X}),$$

where ${\hat{\pi }}_{A}$ is the estimated effect of the genetic variant g on the exposure X in the adjusted GWAS, ${\hat{\pi }}_{C}$ is the estimated effect of g on the covariate C, $\rho_{\mathrm{CX}}$ is the correlation between X and C, the exposure and the covariate in the adjusted GWAS. ${\hat{\pi }}_{X}$ is the estimated effect of g on X from an unadjusted GWAS (Aschard et al. 2015). Expanding this to a set of j genetic variants, denoted j=(1…j), and additionally defining ${\hat{\Gamma }}_{j}$ as the estimated effect of genetic variant j on an outcome of interest Y. Summary data MR estimated using j genetic variants and inverse variance weighting (IVW) estimates $\beta_{t}$, the total effect of the exposure X on the outcome Y in the regression model:

$${\hat{\Gamma }}_{j}=\beta_{t}{\hat{\pi }}_{X,j}+\epsilon_{j}.$$

Replacing ${\hat{\pi }}_{X,j}$ with the estimated effect obtained from the adjusted GWAS (${\hat{\pi }}_{A})$ this becomes;

$${\hat{\Gamma }}_{j}=\beta_{t}^{*}{\hat{\pi }}_{A,j}+\epsilon_{j}$$

$${\hat{\Gamma }}_{j}=\beta_{t}^{*}(-{\hat{\pi }}_{C,j}\rho_{\mathrm{CX}}+{\hat{\pi }}_{X,j})+\epsilon$$

$${\hat{\Gamma }}_{j}=-\beta_{t}^{*}{\hat{\pi }}_{C,j}\rho_{\mathrm{CX}}+\beta_{t}^{*}{\hat{\pi }}_{X,j}+\epsilon$$

For estimation of this model give an unbiased estimate of $\beta_{t}$, that is, for ${\hat{\beta }}_{t}^{*}$ to equal ${\hat{\beta }}_{t},$the term ${\hat{\pi }}_{C,j}\rho_{\mathrm{CX}}$ would need to be zero. This would occur when either only genetic variants that have no effect on the covariate are included in the estimation or there is no correlation between the covariate and the exposure. However, covariates are usually selected because of their correlation with the exposure and so $\rho_{\mathrm{CX}}$ is unlikely to be zero in practice. If genetic variants are selected based on the p‐value for their association with the exposure in the adjusted GWAS, that is, the *p*‐value associated with ${\hat{\pi }}_{A}\;,$as is often the case, then some of the genetic variants selected will have a large value of ${\hat{\pi }}_{C}.$ These genetic variants will give biased estimates of the effect of the exposure on the outcome. The overall bias in the MR estimation will depend on the balance of the genetic variants included in the estimation.

Pleiotropy robust methods may be able to recover unbiased MR estimates if the set of genetic variants overall satisfy the assumptions required. For example, if for the weighted majority of the SNPs ${\hat{\pi }}_{C}=0$ then the weighted median estimator (Bowden et al. 2016) will recover an unbiased estimate of the total effect of the exposure on the outcome. Note that the selection process for the SNPs will induce a negative correlation between ${\hat{\pi }}_{C,j}$ and ${\hat{\pi }}_{X,j}$, even if they are uncorrelated (as one has to be large for the SNP to be selected as an instrument) and so the assumptions required for MR Egger will not be satisfied.

Where possible selecting genetic variants from an unadjusted GWAS will avoid the bias from the adjustment. However, this may also introduce pleiotropy if the covariate is a confounder for the exposure and outcome by selecting genetic variants that act on the exposure through the covariate. Therefore, caution should be applied in the selection of SNPs even when an unadjusted GWAS is available.

Multivariable Mendelian randomisation (MVMR) is an extension of MR that estimates the effect of multiple exposures on an outcome (Burgess and Thompson 2015; Sanderson et al. 2019). MVMR estimates the direct effect of each exposure on the outcome conditional on the other exposures included in the estimation, rather than the total effect as obtained in univariable MR. MVMR can therefore be used to determine whether multiple exposures exert a causal effect on the outcome or one mediates the causal effect of another (Sanderson et al. 2019; Sanderson 2021). MVMR can also estimate direct causal effects of multiple exposures where the genetic variants are thought to have pleiotropic effects through those exposures.

MVMR using inverse variance weighting (IVW) is a weighted estimation of the regression;

$${\hat{\Gamma }}_{j}=\beta_{1}{\hat{\pi }}_{1,j}+\beta_{2}{\hat{\pi }}_{2,j}+\epsilon ,$$

where $\beta_{1}$ is the direct effect of the exposure X on the outcome Y, conditional on the covariate C and $\beta_{2}$ is the direct effect of the covariate C conditional on the exposure X. If our exposures are the adjusted genetic variant‐exposure association and the genetic variant‐covariate association, ${\hat{\pi }}_{A}$ and ${\hat{\pi }}_{C}$, this becomes;

$${\hat{\Gamma }}_{j}=\beta_{1}{\hat{\pi }}_{A,j}+\beta_{2}{\hat{\pi }}_{C,j}+\epsilon$$

$${\hat{\Gamma }}_{j}=\beta_{1}(-{\hat{\pi }}_{C,j}\rho_{\mathrm{CX}}+{\hat{\pi }}_{X,j})+\beta_{2}{\hat{\pi }}_{C,j}+\epsilon$$

$${\hat{\Gamma }}_{j}=\beta_{1}(-{\hat{\pi }}_{C,j}\rho_{\mathrm{CX}})+\beta_{1}{\hat{\pi }}_{X,j}+\beta_{2}{\hat{\pi }}_{C,j}+\epsilon$$

$${\hat{\Gamma }}_{j}=\beta_{1}{\hat{\pi }}_{X,j}+(\beta_{2}{-\beta }_{1}\rho_{\mathrm{CX}}){\hat{\pi }}_{C,j}+\epsilon .$$

Therefore, the estimated effect of the exposure X is the same whether ${\hat{\pi }}_{A}$ or ${\hat{\pi }}_{X}$ is included in the estimation. i.e. MVMR gives the same estimated direct effect of the exposure on the outcome whether the GWAS used is adjusted or unadjusted, if the covariate in the GWAS is included as an exposure in the MVMR. From this expression it can also be seen, that the estimated effect of the covariate when the adjusted GWAS is used will not be the same as the effect estimated from an unadjusted MVMR.

For settings where the outcome GWAS has been adjusted for the covariate rather than the exposure GWAS we can similarly rearrange the terms for the adjusted MVMR to compare them to what would be obtained from MVMR with unadjusted GWAS results;

$${\hat{\Gamma }}_{A,j}=\beta_{1}{\hat{\pi }}_{X,j}+\beta_{2}{\hat{\pi }}_{C,j}+\epsilon$$

$$(-\pi_{C,j}\rho_{\mathrm{CX}}+{\hat{\Gamma }}_{j})=\beta_{1}{\hat{\pi }}_{X,j}+\beta_{2}{\hat{\pi }}_{C,j}+\epsilon$$

$${\hat{\Gamma }}_{j}=\beta_{1}{\hat{\pi }}_{X,j}+\beta_{2}{\hat{\pi }}_{C,j}+{\hat{\pi }}_{C,j}\rho_{\mathrm{CX}}+\epsilon$$

$${\hat{\Gamma }}_{j}=\beta_{1}{\hat{\pi }}_{X,j}+{(\beta }_{2}+\rho_{\mathrm{CX}}){\hat{\pi }}_{C,j}+\epsilon .$$

In a similar way to the adjusted exposure GWAS, incorporating an adjusted outcome GWAS in an MVMR will give the equivalent effect estimate for the exposure of interest but not of the covariate.

In this paper, we investigate the use of MVMR including the covariate that the GWAS for the exposure of interest was adjusted for as an additional exposure. We do this to recover an estimate of the true direct causal effect estimate of the exposure of interest on the outcome when only adjusted GWAS results are available for either the exposure or the outcome. It is important to note that MVMR targets a different causal estimand to MR, the direct effect rather than the total effect. Where the covariate is a mediator of the exposure and outcome these effects will not be the same, however using MVMR in this scenario has the advantage of not imposing any assumptions about the proportion of genetic variants for which ${\hat{\pi }}_{C,j}=0$.

We then apply this method to estimate the causal effect of systolic blood pressure (SBP) on type 2 diabetes (T2D) where the GWAS for SBP has been adjusted for BMI. These results are then compared to those obtained for estimation using GWAS that were not adjusted for BMI.

## 2 Simulation Study

### 2.1 Methods

We performed a simulation study to explore whether it is possible to recover the direct effect of an exposure of interest by including the covariate in a MVMR estimation when the GWAS for either the exposure of interest or outcome has been adjusted for the covariate.

Simulations were performed under three different causal structures in the data for the relationship between the exposure of interest (X_1_), the covariate (X_2_), and the outcome (Y). In each case an unobserved confounder acts on both exposures (X_1,_ X_2_) and the outcome (Y), leading to X_1_ and X_2_ being correlated even when neither has a direct effect on the other. In all simulations X_1_ and X_2_ have a causal effect on Y.

**Figure 1:**
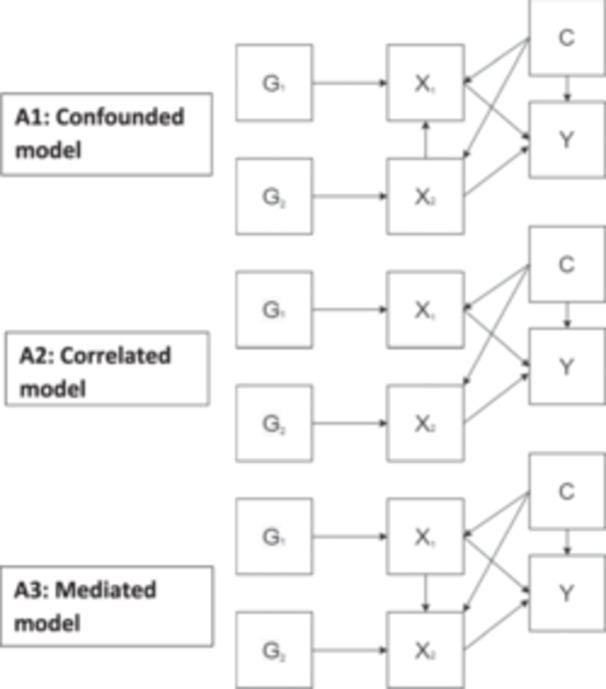
Directed acyclic graphs (DAG) charts displaying the underlying causal structures of the simulated datasets, where G represents simulated variants, X a simulated exposure, Y a simulated outcome, and C an unmeasured common cause.

The first underlying structure (A1 – Confounding) describes a situation where the exposure of interest (X_1_) and outcome (Y) are confounded by a covariate (X_2_), meaning there is a direct causal path from X_2_ to both X_1_ and to Y. This scenario has the potential to bias the estimated effect of X_1_ on Y from MR with unadjusted GWAS summary data due to the identification of variants acting on X_2_ which then influence both X_1_ and Y, introducing pleiotropic variants into the analysis. The second scenario (A2 – Correlation) describes a setting where X_1_ and X_2_ are correlated through shared (unobserved) confounding, this will affect the SNPs identified for X_1_ and X_2_ leading to an increased overlap in genetic instruments. The final causal structure (A3 – Mediation) describes a scenario where X_1_ has an effect on X_2_ as well as Y, meaning X_2_ mediates the X_1_, Y relationship. In this case the direct and total effect of X_1_ on Y will differ as the total effect will include the effect that acts through X_2_. These scenarios are illustrated below (Figure 1).

In each model we generated 250 genetic variants, split equally between $G_{1}$ and $G_{2}$. The effect of each genetic variant in $G_{1}$ on $X_{1}$ was drawn from a normal distribution and similarly the effect of each genetic variant in $G_{2}$ on $X_{2}$ was drawn from a normal distribution. The estimated association between all of the genetic variants (i.e., the combined set of $G_{1}$ and $G_{2}$) and each of the exposure, covariate and exposure adjusted for the covariate were then estimated. The full data‐generating process is described in Supporting Information: Material S1.

MR and MVMR causal estimates were obtained using IVW with summary data for the genetic variant‐exposure and genetic variant‐outcome association. For MR using unadjusted GWAS all genetic variants strongly associated with X were selected as instruments for X, and the effect size from an unadjusted regression of the genetic variant on the exposure was used in the MR estimation. For the MR using adjusted GWAS all genetic variants strongly associated with X in a regression of the genetic variant on the exposure adjusted for the covariate were selected as instruments for X, and the effect size from this regression was used in the MR estimation. For MVMR all genetic variants strongly associated with the covariate were additionally included in the estimation, and the size of the association between the genetic variant and each of the exposure, or adjusted exposure, and covariate were used in the estimation. The association between the genetic variants selected for the MR/MVMR and the outcome were obtained from a regression of each genetic variant on the outcome, either unadjusted or adjusted for the covariate as relevant. F‐statistics were calculated for the MR models, and conditional F‐statistics for each exposure in the multivariable MR models (Sanderson, Spiller, and Bowden 2021). Each simulation had a sample size of 100,000 and 1000 repetitions.

### 2.2 Results

Table 1 gives the IVW univariable MR estimates and coverage of the 95% confidence intervals (CI) from our simulation. These results show estimates of the total effect of the exposure on the outcome with and without adjustment for the covariate ($X_{2}$) in the GWAS for our exposure of interest ($X_{1}$). Without adjustment for the covariate estimates for $\beta_{X1}$ when X2 is a confounder (Model A1) are biased in the direction of $\beta_{X2}$. This is due to a pleiotropic effect of some of the SNPs associated with X_1_ due to some genetic variants associated with X_2_ being detected as associated with X_1_ and so included in the estimation. Estimates for $\beta_{X1}$ when X_2_ is correlated with X_1_ are unbiased estimates of the total effect of X_1_ on Y (Model 2). Estimates for $\beta_{X1}$ when X_2_ is a mediator (Model A3) estimate the total effect of X_1_ on the outcome, including the indirect effect via X_2_. In this setting, the estimated effect of X_2_ is biased in the unadjusted GWAS model as some SNPs associated with X_1_ are selected as instruments for X_2_. When the GWAS for X_1_ is adjusted for X_2_ MR gives biased estimates of the total causal effect of X_1_ on Y in all models. In each case the bias is in the opposite direction to the direct effect of X_2_ on Y ($\beta_{X2})$.

**Table 1:** Simulation results for MR estimation of the effect of X_1_ and X_2_ on Y for, confounded, correlated, and mediated models.

	Unadjusted		Exposure adj.		Outcome adj.		Both adj.	
	X_1_	X_2_	X_1_	X_2_	X_1_	X_2_	X_1_	X_2_
Confounded								
Mean estimate	0.49	0.90	0.18	—	0.49	0.89	0.18	—
Std. error	0.052	0.011	0.074	—	0.053	0.011	0.074	—
Coverage	0.79	0.92	0.00	—	0.79	0.92	0.00	—
* F*‐statistic	222.3	266.3	261.4	—	222.3	266.3	261.4	—
Correlated								
Mean estimate	0.40	0.80	0.20	—	0.40	0.79	0.20	—
Std. error	0.009	0.011	0.067	—	0.009	0.011	0.07	—
Coverage	0.94	0.92	0.00	—	0.94	0.92	0.00	—
* F*‐statistic	300.8	266.3	262.4	—	300.8	266.3	262.4	—
Mediated								
Mean estimate	0.60	0.83	0.22	—	0.60	0.84	0.22	—
Std. error	0.010	0.026	0.080	—	0.010	0.026	0.080	—
Coverage	0.92	0.90	0.00	—	0.93	0.90	0.00	—
* F*‐statistic	300.8	206.6	237.6	—	300.8	206.6	237.6	—

*Note:* Univariable summary data MR estimates for the effect of X_1_ and X_2_ on Y. Total effect of X_1_ in confounded model: 0.4, correlated model: 0.4, mediated model: 0.6. Total effect of X_2_ in confounded model: 0.9, correlated model: 0.8, mediated model: 0.8. Adjustment refers to setting where the GWAS for exposure/outcome has been adjusted for X_2_. All genetic variants for exposure are selected based on association in the exposure estimation sample. *N* = 100,000 reps = 1000.

Abbreviations: GWAS, Genome‐Wide Association studies; MR, Mendelian randomisation.

Table 2 gives MVMR estimates of the direct effect of X_1_ and X_2_ on Y with and without adjustment for X_2_ in the GWAS for X_1_ association. These results give unbiased estimates of the direct effect of X_1_ on Y in both the unadjusted GWAS and adjusted GWAS estimation. However, when the effect of the SNPs adjusted for X_2_ are used in the MVMR the estimated effect of X_2_ on the outcome is biased. These results support the theoretical results given above.

**Table 2:** Simulation results for multivariable MR estimation of the effect of X1 and X_2_ on Y for, confounded, correlated, and mediated models.

	Unadjusted		Exposure adj.		Outcome adj.		Both adj.	
	X_1_	X_2_	X_1_	X_2_	X_1_	X_2_	X_1_	X_2_
Confounded								
Mean estimate	0.39	0.79	0.39	1.01	0.39	0.79	0.39	1.01
Std. error	0.012	0.012	0.012	0.012	0.012	0.012	0.012	0.012
Coverage	0.92	0.91	0.93	0.00	0.92	0.91	0.93	0.00
*F*‐statistic	154.9	159.0	115.3	114.6	154.9	159.0	115.3	114.6
Correlated								
Mean estimate	0.39	0.79	0.40	0.91	0.39	0.79	0.40	0.91
Std. error	0.011	0.011	0.011	0.011	0.011	0.011	0.011	0.012
Coverage	0.92	0.88	0.92	0.00	0.92	0.88	0.92	0.00
*F*‐statistic	153.6	131.7	151.8	129.9	153.6	131.7	151.8	129.9
Mediated								
Mean estimate	0.40	0.79	0.40	0.96	0.40	0.79	0.40	0.96
Std. error	0.012	0.012	0.013	0.012	0.013	0.012	0.012	0.012
Coverage	0.93	0.88	0.93	0.00	0.93	0.88	0.93	0.00
*F*‐statistic	187.9	133.7	133.8	104.7	187.9	133.7	133.8	104.7

*Note:* Multivariable summary data MR estimates for the effect of X_1_ and X_2_ on Y. Direct effect of X_1_ in confounded model: 0.4, correlated model: 0.4, mediated model: 0.4. Direct effect of X_2_ in confounded model: 0.8, correlated model: 0.8, mediated model: 0.8. Adjustment refers to setting where the GWAS for exposure/outcome has been adjusted for X_2_. All genetic variants for exposure are selected based on association in the exposure estimation sample. *N* = 100,000 reps = 1000.

Abbreviations: GWAS, Genome‐Wide Association studies; MR, Mendelian randomisation.

To test the effects of covariate adjustment when the true effect of either the exposure or covariate is null, $\beta_{X1}$ and $\beta_{X2}$ were alternatively set to 0. When $\beta_{X1}=0$ the IVW MVMR accurately estimated the effect of X_2_, even when used as an adjustment for X_1_. When $\beta_{X2}=0$ this pattern was not repeated, the estimated effect of X_2_ was biased as in our main analyses. These results are reported in Supporting Information: Tables 1 and 2. We additionally explored the results obtained when X_1_ and X_2_ act in opposite directions on the outcome. These results showed the same pattern of result as obtained in the main analysis and are given in Supporting Information: Tables 3 and 4.

Tables 1 and 2 also show the results from MR and MVMR estimation and coverage of the 95% CIs of the same models when the GWAS for the outcome, Y, is adjusted for a covariate. These results showed little effect of bias due to the outcome adjustment, with similar estimates to that of the unadjusted estimates. As SNPs are selected based on their association with the exposure, when the exposure has not been adjusted the number of SNPs with an association with X_2_ included in the estimation is minimal and so bias is not observed in the MR estimates.

F‐statistics and conditional F‐statistics were calculated for each simulation and averaged across the iterations (Sanderson, Spiller, and Bowden 2021), these were large for all of the estimation models suggesting that the results obtained are unlikely to be biased by weak instruments.

Coverage in all of the unbiased estimations is between 92% and 94% in the MR estimation and between 88% and 92% in the MVMR estimation. This is much higher than for the biased MR estimation using the adjusted exposure data (which has a coverage of 0%). It is however, slightly lower than the nominal level. This is likely to be because the SNPs used as instruments in the simulation have been selected based on their association with the exposure in the same data used for the exposure in the MR or MVMR estimation, introducing an element of winners curse to the results. Additionally, as the F‐statistics reported are the mean across all of the iterations of the simulation it is possible that the instruments were weak in some individual iterations. This would bias the effect estimates obtained in that iteration away from the mean and potentially lower the overall coverage in the simulations.

These results show how univariable MR effect estimates are biased when SNP‐trait associations with the exposure are obtained from a covariate‐adjusted GWAS. However, use of covariate‐adjusted GWAS as an outcome in MR appears to introduce less bias. As shown earlier MR is only biased when SNPs associated with the covariate are included in the estimation (i.e., $\pi_{C}\neq 0)$. As the SNPs here have been selected to be those associated with the exposure and we have not included pleiotropic effects in the model such association will only potentially occur when the covariate is a confounder of the exposure and has a strong enough effect on the exposure that SNPs associated with C (and so indirectly the exposure) are strongly enough associated with the exposure to be selected as instruments for the exposure. In these simulations, very few SNPs are likely to be associated with the exposure in such a way and so the MR estimates obtained are unbiased. MVMR, including the adjustment covariate recover an unbiased estimate of the direct effect of the exposure of interest (X_1_), providing confirmation that the MR results were unbiased, but does not provide an unbiased estimate of the covariate on the outcome.

## 3 Application

### 3.1 Methods

To illustrate this method we considered two applications using traits that are often adjusted for body mass index (BMI). We estimate the causal effect of systolic blood pressure (SBP) on type‐2 diabetes (T2D) using both MR and MVMR with GWAS summary results. We then considered the estimation of the effect of waist circumference (WC) on SBP where either, or both, of the GWAS for WC or SBP were adjusted for BMI.

To estimate the effect of SBP on T2D we used summary data for BMI and SBP from an unadjusted GWAS using data from UK Biobank and hosted on OpenGWAS (Elsworth et al. 2020), while the GWAS study adjusted for BMI used data from UK Biobank and the International Consortium of Blood Pressure‐Genome Wide Association Studies (ICBP) (Evangelou et al. 2018). GWAS results for T2D, unadjusted for BMI, were obtained from the 70K for T2D GWAS to avoid overlap with UK Biobank (Bonàs‐Guarch et al. 2018a).

To estimate the effect of WC on SBP we used GWAS summary data for WC from a GIANT study, this data was available both unadjusted and adjusted for BMI (Shungin et al. 2015). We used the same unadjusted SBP GWAS summary‐statistics as in the first example (Elsworth et al. 2020). BMI GWAS summary statistics were obtained from the GIANT cohort (Yengo et al. 2018).

The causal effects were estimated using MR‐IVW and MVMR‐IVW, genetic variants were selected on the basis that they were robustly associated with the exposure in a GWAS of that exposure, based on a genome‐wide significant *p*‐value < 5e−08. Each study applied its own quality control, details of which can be found in the original publications (Evangelou et al. 2018; Shungin et al. 2015; Elsworth et al. 2020; Yengo et al. 2018; Bonàs‐Guarch et al. 2018b). Genome‐wide significant SNPs were clumped to remove SNPs in LD with each other (*R* ^2^ threshold for considering LD was set to 0.001). Summary association data for the exposure and outcome for the selected SNPs were harmonised to reflect the same effect alleles and palindromic SNPs with intermediate allele frequencies were excluded. For MR estimation we also conducted MR‐Egger (Bowden, Davey Smith, and Burgess 2015), Weighted Mode (Hartwig, Davey Smith, and Bowden 2017) and Weighted median (Bowden et al. 2016) estimation to assess how sensitive the results were to potential pleiotropy. MR estimation was conducted using the R package ‘TwoSampleMR’ (Hemani et al. 2018) and MVMR estimation with the R package ‘MVMR’ (Sanderson, Spiller, and Bowden 2020).

### 3.2 Results

In the IVW univariable MR analyses of SBP on T2D (Table 3) using unadjusted summary statistics gives a positive causal effect of SBP on T2D (OR increase in T2D of 1.47 per SD increase in SBP, 95% CI: 1.25–1.72). Using SBP GWAS results adjusted for BMI the effect estimate obtained was: 1.36 odds ratio for risk of T2D per SD increase in SBP (95% CI: 1.14–1.64). In the MVMR model including BMI as a covariate the odds ratio per SD increase in SBP is 1.32 (95% CI: 1.10–1.60), when the GWAS for SBP adjusted for BMI was used very similar effect estimates were obtained: OR 1.34 (95% CI: 1.10–1.62) (Table 4). As discussed above, the estimated effects for the covariate in the MVMR are biased estimates of the effect of that covariate on the outcome and so we do not attempt to interpret the effect of BMI in these MVMR models.

**Table 3:** IVW univariable MR effect size estimates SBP and BMI on type 2 diabetes, effect size represents odds ratios per SD increase in SBP.

	Unadjusted estimates				Adjusted estimates	
	SBP		BMI		SBP (adjusted for BMI)	
	OR	95% CI	OR	95% CI	OR	95% CI
IVW	1.47	1.25–1.72	1.41	1.25–1.58	1.36	1.14–1.64
Weighted median	1.25	1.01–1.55	1.46	1.23–1.74	1.07	0.89–1.30
Weighted mode	0.99	0.64–1.53	1.47	1.16–2.11	1.09	0.76–1.56
*F*‐statistic	17.9		63.9		13.1	

*Note:* OR: Estimated odds ratio for Type 2 diabetes per standard deviation increase in systolic blood pressure (SBP). 95% CI, 95% confidence interval for the estimated effect. Effects estimated using 244 single‐nucleotide polymorphisms (SNPs) identified for SBP and 461 for body mass index (BMI), summary data used was obtained from: UK Biobank (SBP unadjusted), International Consortium of Blood Pressure (SBP adjusted) (Evangelou et al. 2018), 70K for T2D (type‐2 diabetes) (Bonàs‐Guarch et al. 2018a), UK Biobank (BMI).

Abbreviations: IVW, inverse variance weighting; MR, Mendelian randomisation; SD, standard deviation.

**Table 4:** IVW MVMR effect size estimates for SBP and BMI on type 2 diabetes, effect size represents odds ratios per SD increase in SBP.

	Unadjusted estimates				Adjusted estimates			
	SBP		BMI		SBP (adjusted for BMI)		BMI	
	OR	95% CI	OR	95% CI	OR	95% CI	OR	95% CI
IVW	1.32	1.10–1.60	1.35	1.19–1.53	1.34	1.10–1.62	1.32	1.12–1.57
Conditional *F*‐statistic	22.1		47.1		53.0		37.0	

*Note:* OR: Estimated odds ratio for Type 2 diabetes. 95% CI, 95% confidence interval for the estimated effect. Effects were estimated using 424 single‐nucleotide polymorphisms (SNPs) identified across both systolic blood pressure (SBP) and body mass index (BMI) for unadjusted estimates, and 490 for adjusted estimates, summary data used was obtained from: UK Biobank (SBP unadjusted), International Consortium of Blood Pressure (SBP adjusted) (Evangelou et al. 2018), 70K for T2D (type‐2 diabetes) (Bonàs‐Guarch et al. 2018a), UK Biobank (BMI).

Abbreviations: IVW, inverse variance weighting; MR, Mendelian randomisation; SD, standard deviation.

Sensitivity analyses for the MR estimation showed inconsistent results for both unadjusted and adjusted SBP. This potentially reflects pleiotropic effects due to BMI confounding the SBP‐T2D association and SNPs associated with BMI primarily being detected in the unadjusted SBP GWAS and incorrectly adjusted for in the adjusted GWAS results (Sanderson et al. 2024).

These same MVMR analyses, to estimate the effect of SBP and BMI on T2D, were repeated instead using only using the SNPs identified as being associated with SBP in the SBP GWAS (Evangelou et al. 2018). This was done to explore whether the differences between the obtained results were due to differing numbers of SNPs in each analysis. Results are given in Supporting Information: Tables 5 and 6. The estimates using both the unadjusted and adjusted sample data were very similar to the full SNP analyses.

Table 5 gives the results from the estimation of the effect of WC on SBP. The effect of waist circumference on SBP was estimated as a small increase in SBP (0.0359 mmHg per SD increase in WC, 95% CI: 0.031–0.103) using summary data unadjusted for the covariate BMI. When using GWAS results for WC adjusted for BMI a slight decrease in SBP due to WC was estimated (−0.062 95% CI: −0.138 to 0.001). Using GWAS results for the outcome SBP adjusted for BMI the estimated effect was negative like the estimates produced by the analyses using a covariate‐adjusted exposure (−0.081 95% CI: −0.145 to −0.016). Here the MR Egger, weighted median and weighted mode estimates had roughly the same effect size and magnitude as the IVW estimates across all analyses.

**Table 5:** IVW univariable MR estimates for effect size of Waist circumference on SBP, effect size represents increase in SBP per SD increase in WC.

	Unadjusted estimates				Adjusted exposure estimates		Adjusted outcome estimates	
	WC		BMI		WC (adjusted for BMI)		WC (where SBP is adjusted for BMI)	
	*β*	95% CI	*β*	95% CI	*β*	95% CI	*β*	95% CI
IVW	0.036	0.031–0.103	0.122	0.095–0.148	−0.062	−0.138 to 0.001	−0.081	−0.145 to −0.016
MR‐Egger	0.126	−0.112 to 0.363	0.096	0.0266–0.166	−0.069	−0.354 to 0.231	−0.118	−0.346 to 0.111
Weighted median	0.077	0.027–0.128	0.140	0.113–0.168	−0.036	−0.078 to 0.005	−0.007	−0.046 to 0.033
Weighted mode	0.103	−0.019 to 0.225	0.160	0.105–0.215	−0.042	−0.118 to 0.034	−0.007	−0.063 to 0.049
*F*‐statistic	70.7		72.84		45.7		70.7	

*Note: β*: Estimated effect size for systolic blood pressure (SBP). 95% CI, 95% confidence interval for the estimated effect. Effect sizes were estimated using 42 SNPs identified for WC in unadjusted estimates, 65 in adjusted estimates and 461 for BMI. GWAS summary data was obtained from GIANT (WC), UK Biobank (BMI, SBP unadjusted), and International Consortium of Blood Pressure (SBP adjusted).

Abbreviations: BMI, body mass index; GWAS, Genome‐Wide Association studies; IVW, inverse variance weighting; MR, Mendelian randomisation; SD, standard deviation; SNPs, single‐nucleotide polymorphisms; WC, waist circumference.

There is a notable difference in the interpretation of the results here with and without adjustment for BMI, however, it was not possible to apply MVMR to obtain an unbiased estimate of the direct effect of WC on SBP in this setting as the conditional *F*‐statistics were too low to estimate the model. In the model using unadjusted WC and BMI the conditional *F*‐statistics were 0.9 for WC and 1.0 for BMI. In the estimation using adjusted WC and BMI they were 3.2 and 64.8, respectively. Therefore, even when we have included adjusted WC there is insufficient statistical variation in the model to strongly estimate the effect of WC on SBP.

## 4 Discussion

Covariate‐adjusted GWAS results can bias the results from MR studies when either the exposure or outcome has been adjusted for a covariate (Hartwig et al. 2021; Walker et al. 2021). Here, we highlight how MR is biased by covariate adjustment and show that MVMR can be used to obtain unbiased direct causal effect estimates from covariate‐adjusted GWAS studies by including the covariate as an additional exposure. The estimated results obtained are the direct effect of the exposure on the outcome, conditional on the covariate. When the covariate is a mediator of the exposure and outcome this will differ from the target estimand for MR. It is important to note that MVMR cannot be used to provide an accurate estimate of the causal effect of the covariate on the outcome when either the exposure or the outcome has been adjusted for the covariate.

Using simulated data we found there were expected differences in IVW estimates across univariable MR estimates using unadjusted GWAS summary statistics under different simulated causal structures. When the covariate was a confounder of the exposure and outcome relationship we additionally observed bias because of pleiotropic effects of genetic variants associated with that covariate being included in the MR estimation. When the covariate was a mediator the total effect estimated in the unadjusted univariable estimates was higher than the direct effect due to the effect of the mediator on the outcome. When using the exposure summary statistics adjusted for the covariate the univariable MR IVW estimated effect of the exposure of interest was biased in the opposite direction to the true effect of the covariate on the outcome (Table 1). This was as expected due to the bias in the SNPs exposure association introduced by the adjustment, creating spurious associations between SNPs and the exposure and an inverse relationship between adjusted exposure and SNPs associated with the covariate. The reduced bias observed in scenarios where the outcome is adjusted for the covariate is likely due to the lack of bias induced by differential SNP selection as instruments are selected by association with the exposure for univariable MR. Consequently, SNPS associated with the covariate were not included in the estimation and so there was no bias in the MR results even with the adjusted outcome GWAS. When analysing the same simulated data sets in MVMR the estimates obtained were unbiased even with the covariate adjustments (Table 2).

Our real data applied examples support the results in the simulation. In our SBP‐T2D example, the MR estimates showed a small potential difference between the causal effect estimate using data from the adjusted and unadjusted GWAS in the opposite direction to that of the effect direction of the covariate (BMI) on the outcome. The variation in the effect estimates obtained by the sensitivity analyses suggests that substantial horizontal pleiotropy was present. As highlighted earlier, heterogeneity in the MR estimates is likely due to the bias in the MR study induced by the adjustment in the GWAS. Some pleiotropy robust estimation methods are likely to be robust to this bias if the SNPs selected satisfy the assumption for that method. The MVMR estimates for both adjusted and unadjusted summary data were very similar to each other. As with our findings displayed here previous observational and MR studies have identified a positive correlation and associations between systolic blood pressure and T2D (Aikens et al. 2017; Wei et al. 2011).

Our second example considered the estimation of the effect of WC on SBP. In this case the results for the MR using GWAS for WC that were adjusted or unadjusted for BMI lead to notably different interpretation of the results, with effect estimates in opposite directions and broadly consistent results across the range of sensitivity analyses applied in each case. These results highlight the importance of considering adjustment in the GWAS. We were unable to apply MVMR correction in this setting due to weak instruments in the MVMR models.

Our study proposes a simple solution to the issue of bias in MR estimation from covariate adjustment in the GWAS. MVMR with summary‐level data is already widely implemented across a range of applications, and therefore existing data sources and software can be easily extended to use this method. However, an important limitation of our proposed solution is that this approach can only estimate the direct effect of the exposure of interest on the outcome. This effect may differ from the total effect if the covariate is a mediator of the exposure outcome relationship and our proposed method does not provide an approach to recover the total effect of the exposure on the outcome. Estimation also requires summary GWAS results for the covariate to be available and for the genetic variants to be conditionally strong enough to obtain reliable estimates from the MVMR model.

Another limitation of this method is that the estimate of the direct effect of the covariate on the outcome obtained from this approach is potentially biased and cannot, therefore, be interpreted in the same way as a conventional MVMR analysis would. This is a particularly important limitation as the method will produce estimates of these effects but it is important that they are not interpreted as they are not estimates of the direct effect of the covariate on the outcome.

Finally, this method requires that all of the standard assumptions of MVMR, such as no weak instruments hold. These assumptions can be tested in the same way as any standard MVMR estimation (Sanderson, Spiller, and Bowden 2020; Wei et al. 2011). However, if the exposure and covariate are closely related, or one of the adjusted exposure and covariate have only a few SNPs associated with them then it is more likely that the MVMR will be biased by weak instruments. Consequently this method of correction would not be able to be applied. This was highlighted by our second application where it was not possible to reliably estimate the model by MVMR.

In the case where a choice between using a non‐adjusted GWAS and an adjusted GWAS with the methodology laid out in this paper is available the non‐adjusted GWAS is still preferable. First, non‐adjusted GWAS allow for unbiased estimates of the total effect size (as opposed to the direct effect size estimated with MVMR). Second, when estimating effect sizes using MVMR with a non‐adjusted GWAS the effect size estimates from the other exposures can be interpreted, something not possible when including the covariate in MVMR with an adjusted GWAS.

In conclusion, when using covariate‐adjusted summary association results from a GWAS the bias that the covariate introduces may be overcome by using a MVMR approach where the covariate used to adjusted the GWAS is included in the analysis as a second exposure.

## Author Contributions

Joe Gilbody conducted the simulation study and applied analysis and wrote the first draft of the manuscript. Eleanor Sanderson developed the methodology, supervised the work and contributed to drafting the manuscript. All authors reviewed and edited the manuscript.

## Ethics Statement

All data analysed were from publicly available summary statistics generated using relevant ethical approval from their respective studies.

## Conflicts of Interest

The authors declare no conflicts of interest.

## Supporting information

Supporting information.

## Data Availability

The data that support the findings of this study are available in Git repository at https://github.com/eleanorsanderson/adjustedGWAS. These data were derived from the following resources available in the public domain: ‐ Open GWAS, https://gwas.mrcieu.ac.uk/.
