# Supplementary material for: Multivariable MR Can Mitigate Bias in Two‐Sample MR Using Covariable‐Adjusted Summary Associations
Source: Genet Epidemiol. 2025 Jan 15;49(1):e22606. doi: 10.1002/gepi.22606 (PMC11734645; doi:10.1002/gepi.22606)
Supplement: Supplementary file 1 — Supporting information. [file GEPI-49-0-s001.docx]

**Multivariable MR can mitigate bias in two-sample MR using covariable-adjusted summary associations**

**SUPPLEMENTARY MATERIAL**

**Joe Gilbody*, Maria Carolina Borges, George Davey Smith, Eleanor Sanderson**

*MRC Integrative Epidemiology Unit, University of Bristol, Bristol, UK*

*Population Health Sciences, University of Bristol, Bristol, UK*

**Corresponding author: joe.gilbody@bristol.ac.uk*

**SECTION 1 – Additional Simulation details**

**1.1 Additional details of the simulations**

The data generating models for the three scenarios were defined by the following expressions;

Confounded:

$$X_{1}= \pi_{1}G+0.25X_{2}+0.8C+v_{1}$$

$$X_{2}=\pi_{2}G+C+v_{2}$$

$$Y= \beta_{1}X_{1}+\beta_{2}X_{2}+0.5C+v_{y}$$

Correlated:

$$X_{1}= \pi_{1}G+0.8C+v_{1}$$

$$X_{2}=\pi_{2}G+C+v_{2}$$

$$Y= \beta_{1}X_{1}+\beta_{2}X_{2}+0.5C+v_{y}$$

Mediated:

$$X_{1}= \pi_{1}G+0.8C+v_{1}$$

$$X_{2}=\pi_{2}G+0.25X_{1}+C+v_{2}$$

$$Y= \beta_{1}X_{1}+\beta_{2}X_{2}+0.5C+v_{y}$$

Where $G$ is a set of 400 genetic variants drawn from a binomial distribution with a mean minor allele frequency of 0.4. $\pi_{1}$and $\pi_{2}$are sets of effects of those genetic variants on $X_{1}$and $X_{2}$ respectively. $\pi_{1}$and $\pi_{2}$ each have 200 non-overlapping zero elements so each genetic variant affects either one of $X_{1}$or $X_{2}$ but not both. $C$ is a normally distributed confounder of $X_{1}$, $X_{2}$ and $Y$. $v_{1}$, $v_{2}$ and $v_{y}$ are independent randomly normally distributed variables.

To generate the summary statistics for MR estimation the association between $X_{1}$ and all 400 genetic variants was estimated, with or without adjustment for $X_{2}$ as appropriate. Those variants that were associated with $X_{1}$ were then included in the MR estimation. A second independent sample was generated with the same data generating process from which the association between the selected variants and $Y$ was obtained.

**Supplementary Table 1 – Simulation results for MR estimation of the effect of X1 and X_2_ on Y for, confounded, correlated and mediated models with null effect of exposure on the outcome.**

|  | **Unadjusted** | | **Exposure adj.** | | **Outcome adj.** | | **Both adj.** | |
| --- | --- | --- | --- | --- | --- | --- | --- | --- |
|  | **X_1_** | **X_2_** | **X_1_** | **X_2_** | **X_1_** | **X_2_** | **X_1_** | **X_2_** |
| **Confounded** |  |  |  |  |  |  |  |  |
| $\hat{\beta}$ | 0.06 | 0.50 | -0.11 |  | 0.06 | 0.50 | -0.11 |  |
| *Std. Error* | 0.033 | 0.007 | 0.034 |  | 0.033 | 0.007 | 0.034 |  |
| *Coverage* | 0.73 | 0.93 | 0.00 |  | 0.73 | 0.93 | 0.00 |  |
| *F-statistic* | 222.3 | 266.3 | 261.4 |  | 222.3 | 266.3 | 261.4 |  |
| **Correlated** |  |  |  |  |  |  |  |  |
| $\hat{\beta}$ | 0.00 | 0.50 | -0.11 |  | 0.00 | 0.50 | -0.11 |  |
| *Std. Error* | 0.006 | 0.007 | 0.036 |  | 0.006 | 0.007 | 0.036 |  |
| *Coverage* | 0.96 | 0.93 | 0.00 |  | 0.96 | 0.93 | 0.00 |  |
| *F-statistic* | 300.8 | 266.3 | 261.4 |  | 300.8 | 266.3 | 261.4 |  |
| **Mediated** |  |  |  |  |  |  |  |  |
| $\hat{\beta}$ | 0.12 | 0.50 | -0.09 |  | 0.12 | 0.50 | -0.09 |  |
| *Std. Error* | 0.007 | 0.007 | 0.041 |  | 0.007 | 0.007 | 0.041 |  |
| *Coverage* | 0.95 | 0.90 | 0.00 |  | 0.95 | 0.90 | 0.00 |  |
| *F-statistic* | 300.8 | 206.6 | 237.6 |  | 300.8 | 206.6 | 237.6 |  |

Univariable summary data MR estimates for the effect of X_1_ and X_2_ on Y. Total effect of X_1_ in confounded model: 0.0, correlated model: 0.0, mediated model: 0.125. Total effect of X_2_ in confounded model: 0.5, correlated model: 0.5, mediated model: 0.5. Adjustment refers to setting where the GWAS for exposure/outcome has been adjusted for X_2_. All genetic variants for exposure are selected based on association in the exposure estimation sample. N=100,000 reps = 1000.

**Supplementary Table 2 – Simulation results for MR estimation of the effect of X1 and X_2_ on Y for, confounded, correlated and mediated models with null effect of exposure on outcome.**

|  | **Unadjusted** | | **Exposure adj.** | | **Outcome adj.** | | **Both adj.** | |
| --- | --- | --- | --- | --- | --- | --- | --- | --- |
|  | **X_1_** | **X_2_** | **X_1_** | **X_2_** | **X_1_** | **X_2_** | **X_1_** | **X_2_** |
| **Confounded** |  |  |  |  |  |  |  |  |
| $\hat{\beta}$ | -0.00 | 0.50 | -0.00 | 0.50 | -0.00 | 0.50 | -0.00 | 0.50 |
| *Std. Error* | 0.007 | 0.007 | 0.007 | 0.007 | 0.007 | 0.007 | 0.007 | 0.007 |
| *Coverage* | 0.94 | 0.92 | 0.95 | 0.93 | 0.94 | 0.91 | 0.95 | 0.90 |
| *F-statistic* | 154.9 | 159.0 | 115.2 | 114.6 | 154.9 | 159.0 | 115.2 | 114.6 |
| **Correlated** |  |  |  |  |  |  |  |  |
| $\hat{\beta}$ | -0.00 | 0.50 | -0.00 | 0.50 | -0.00 | 0.50 | -0.00 | 0.50 |
| *Std. Error* | 0.007 | 0.007 | 0.007 | 0.007 | 0.007 | 0.007 | 0.007 | 0.007 |
| *Coverage* | 0.95 | 0.91 | 0.95 | 0.90 | 0.95 | 0.91 | 0.95 | 0.90 |
| *F-statistic* | 153.6 | 131.7 | 151.8 | 129.9 | 153.6 | 131.7 | 151.8 | 129.9 |
| **Mediated** |  |  |  |  |  |  |  |  |
| $\hat{\beta}$ | -0.00 | 0.50 | -0.00 | 0.50 | -0.00 | 0.50 | -0.00 | 0.50 |
| *Std. Error* | 0.008 | 0.008 | 0.008 | 0.008 | 0.008 | 0.008 | 0.008 | 0.008 |
| *Coverage* | 0.94 | 0.90 | 0.95 | 0.89 | 0.94 | 0.90 | 0.95 | 0.89 |
| *F-statistic* | 1887.9 | 133.7 | 133.8 | 104.7 | 1887.9 | 133.7 | 133.8 | 104.7 |

Multivariable summary data MR estimates for the effect of X_1_ and X_2_ on Y. Direct effect of X_1_ in confounded model: 0.0, correlated model: 0.0, mediated model: 0.0. Direct effect of X_2_ in confounded model: 0.5, correlated model: 0.5, mediated model: 0.5. Adjustment refers to setting where the GWAS for exposure/outcome has been adjusted for X_2_. All genetic variants for exposure are selected based on association in the exposure estimation sample. N=100,000 reps = 1000.

**Supplementary Table 3 – Simulation results for MR estimation of the effect of X1 and X_2_ on Y for, confounded, correlated and mediated models.**

|  | **Unadjusted** | | **Exposure adj.** | | **Outcome adj.** | | **Both adj.** | |
| --- | --- | --- | --- | --- | --- | --- | --- | --- |
|  | **X_1_** | **X_2_** | **X_1_** | **X_2_** | **X_1_** | **X_2_** | **X_1_** | **X_2_** |
| **Confounded** |  |  |  |  |  |  |  |  |
| $\hat{\beta}$ | 0.30 | -0.70 | 0.52 |  | 0.30 | -0.70 | 0.52 |  |
| *Std. Error* | 0.05 | 0.007 | 0.04 |  | 0.05 | 0.007 | 0.04 |  |
| *Coverage* | 0.69 | 0.88 | 0.00 |  | 0.69 | 0.88 | 0.00 |  |
| *F-statistic* | 222.3 | 266.3 | 261.4 |  | 222.3 | 266.3 | 261.4 |  |
| **Correlated** |  |  |  |  |  |  |  |  |
| $\hat{\beta}$ | 0.40 | -0.80 | 0.54 |  | 0.40 | -0.80 | 0.54 |  |
| *Std. Error* | 0.006 | 0.008 | 0.049 |  | 0.006 | 0.008 | 0.049 |  |
| *Coverage* | 0.94 | 0.88 | 0.00 |  | 0.94 | 0.88 | 0.00 |  |
| *F-statistic* | 300.8 | 266.3 | 261.4 |  | 300.8 | 266.3 | 261.4 |  |
| **Mediated** |  |  |  |  |  |  |  |  |
| $\hat{\beta}$ | 0.20 | -0.75 | 0.51 |  | 0.20 | -0.75 | 0.51 |  |
| *Std. Error* | 0.006 | 0.027 | 0.051 |  | 0.006 | 0.027 | 0.051 |  |
| *Coverage* | 0.94 | 0.64 | 0.00 |  | 0.94 | 0.64 | 0.00 |  |
| *F-statistic* | 300.8 | 206.6 | 237.6 |  | 300.8 | 206.6 | 237.6 |  |

Univariable summary data MR estimates for the effect of X_1_ and X_2_ on Y. Total effect of X_1_ in confounded model: 0.4, correlated model: 0.4, mediated model: 0.2. Total effect of X_2_ in confounded model: -0.7, correlated model: 0.8, mediated model: 0.8. Adjustment refers to setting where the GWAS for exposure/outcome has been adjusted for X_2_. All genetic variants for exposure are selected based on association in the exposure estimation sample. N=100,000 reps = 1000.

**Supplementary Table 4 – Simulation results for multivariable MR estimation of the effect of X1 and X_2_ on Y for, confounded, correlated and mediated models.**

|  | **Unadjusted** | | **Exposure adj.** | | **Outcome adj.** | | **Both adj.** | |
| --- | --- | --- | --- | --- | --- | --- | --- | --- |
|  | **X_1_** | **X_2_** | **X_1_** | **X_2_** | **X_1_** | **X_2_** | **X_1_** | **X_2_** |
| **Confounded** |  |  |  |  |  |  |  |  |
| $\hat{\beta}$ | 0.40 | -0.79 | 0.40 | -0.57 | 0.40 | -0.79 | 0.40 | -0.57 |
| *Std. Error* | 0.007 | 0.008 | 0.007 | 0.008 | 0.007 | 0.008 | 0.007 | 0.008 |
| *Coverage* | 0.95 | 0.88 | 0.95 | 0.00 | 0.95 | 0.86 | 0.95 | 0.00 |
| *F-statistic* | 154.9 | 159.0 | 115.3 | 114.6 | 154.9 | 159.0 | 115.3 | 114.6 |
| **Correlated** |  |  |  |  |  |  |  |  |
| $\hat{\beta}$ | 0.40 | -0.79 | 0.40 | -0.67 | 0.40 | -0.79 | 0.40 | -0.67 |
| *Std. Error* | 0.008 | 0.008 | 0.008 | 0.008 | 0.008 | 0.008 | 0.008 | 0.008 |
| *Coverage* | 0.94 | 0.85 | 0.94 | 0.00 | 0.94 | 0.85 | 0.94 | 0.00 |
| *F-statistic* | 153.6 | 131.9 | 151.8 | 129.9 | 153.6 | 131.9 | 151.8 | 129.9 |
| **Mediated** |  |  |  |  |  |  |  |  |
| $\hat{\beta}$ | 0.40 | -0.79 | 0.40 | -0.62 | 0.40 | -0.79 | 0.40 | -0.62 |
| *Std. Error* | 0.008 | 0.008 | 0.008 | 0.008 | 0.008 | 0.008 | 0.008 | 0.008 |
| *Coverage* | 0.94 | 0.84 | 0.94 | 0.00 | 0.94 | 0.84 | 0.94 | 0.00 |
| *F-statistic* | 187.9 | 133.7 | 133.7 | 104.7 | 187.9 | 133.7 | 133.7 | 104.7 |

Multivariable summary data MR estimates for the effect of X_1_ and X_2_ on Y. Direct effect of X_1_ in confounded model: 0.4, correlated model: 0.4, mediated model: 0.4. Direct effect of X_2_ in confounded model: -0.8, correlated model: -0.8, mediated model: -0.8. Adjustment refers to setting where the GWAS for exposure/outcome has been adjusted for X_2_. All genetic variants for exposure are selected based on association in the exposure estimation sample. N=100,000 reps = 1000.

**SECTION 2 – Additional results for applications**

**Supplementary Table 5. IVW two-sample MR estimates of SBP and BMI on Type 2 diabetes using only SNPs found to be associated with SBP, effect size represent odds ratios per SD increase in SBP**

|  | **Unadjusted estimates** | | | | **Adjusted Estimates** | | | |
| --- | --- | --- | --- | --- | --- | --- | --- | --- |
|  | **SBP OR** | **Confidence intervals** | **BMI OR** | **Confidence intervals** | **SBP OR** | **Confidence intervals** | **BMI OR** | **Confidence intervals** |
| **IVW** | 1.47 | 1.25 – 1.71 | 0.76 | 0.19 – 3.13 | 1.11 | 1.04- 1.19 | 0.70 | 0.48 – 1.03 |
| Effect sizes estimated using 122 SNPs identified for SBP, , summary data used was obtained from: UK Biobank (SBP unadjusted), International Consortium of Blood Pressure (SBP adjusted) (8), 70KforT2D (type-2 diabetes) (16), UK Biobank (BMI). | | | | | | | | |

**Supplementary Table 6. IVW MVMR estimates of SBP and BMI on type 2 diabetes using only SNPs found to be associated with SBP, effect size represent odds ratios per SD increase in SBP**

|  | **Unadjusted estimates** | | | | **Adjusted Estimates** | | | |
| --- | --- | --- | --- | --- | --- | --- | --- | --- |
|  | **SBP OR** | **Confidence intervals** | **BMI OR** | **Confidence intervals** | **SBP OR** | **Confidence intervals** | **BMI OR** | **Confidence intervals** |
| **IVW** | 1.33 | 1.09-1.60 | 0.74 | 0.39 – 1.40 | 1.26 | 1.01 – 1.57 | 0.55 | 0.30 – 1.01 |
| Effect sizes estimated using 284 SNPs identified for SBP, summary data used was obtained from: UK Biobank (SBP unadjusted), International Consortium of Blood Pressure (SBP adjusted) (8), 70KforT2D (type-2 diabetes) (16), UK Biobank (BMI). | | | | | | | | |
